# Meta‐Analysis: Inverse Association Between *Helicobacter pylori* Infection and Eosinophilic Oesophagitis

**DOI:** 10.1111/apt.70042

**Published:** 2025-02-24

**Authors:** Irene Spinelli, Serena Porcari, Chiara Esposito, William Fusco, Francesca Romana Ponziani, Cristiano Caruso, Edoardo Vincenzo Savarino, Antonio Gasbarrini, Giovanni Cammarota, Marcello Maida, Antonio Facciorusso, Gianluca Ianiro

**Affiliations:** ^1^ Department of Translational Medicine and Surgery Università Cattolica del Sacro Cuore Rome Italy; ^2^ Department of Medical and Surgical Sciences, UOC CEMAD Centro Malattie dell'Apparato Digerente, Medicina Interna e Gastroenterologia Fondazione Policlinico Universitario Agostino Gemelli IRCCS Rome Italy; ^3^ Department of Medical and Surgical Sciences, UOC Gastroenterologia Fondazione Policlinico Universitario Agostino Gemelli IRCCS Rome Italy; ^4^ UOSD Allergologia e Immunologia Clinica Fondazione Policlinico Universitario Agostino Gemelli IRCCS Rome Italy; ^5^ Department of Surgery, Oncology and Gastroenterology University of Padova Padova Italy; ^6^ Gastroenterology Unit Azienda Ospedale Università di Padova Padova Italy; ^7^ Department of Medicine and Surgery University of Enna ‘Kore’ Enna Italy; ^8^ Gastroenterology Unit Umberto I Hospital Enna Italy; ^9^ Department of Experimental Medicine Università del Salento Lecce Italy; ^10^ Clinical Effectiveness Research Group University of Oslo Oslo Norway

**Keywords:** eosinophilic esophagitis, *Helicobacter pylori*, meta‐analysis

## Abstract

**Background:**

Exposure to *Helicobacter pylori* ( *H. pylori* ) has been associated with a decreased risk of eosinophilic oesophagitis (EoE).

**Aim:**

The aim of this study is to determine the association between *H. pylori* infection and EoE in this updated meta‐analysis.

**Methods:**

We searched MEDLINE, Scopus and ISI Web of Science, through to November 2024. We included studies reporting the status of *H. pylori* infection in patients with and without EoE or oesophageal eosinophilia (EE). We used a random‐effects model to pool estimates.

**Results:**

We analysed 19 studies including 1.704.821 subjects. *H. pylori* infection was associated with a 46% lower risk of EoE/EE (OR: 0.54, 95% CI 0.43 to 0.67). Comparable findings were observed when subgrouping studies by location or design. There was a nonsignificant decrease in odds for EoE in paediatric patients exposed to *H. pylori* (OR 0.57, 95% CI 0.26 to 1.24), and in studies using serology to diagnose *H. pylori* (OR: 0.41, 95% CI 0.16 to 1.04). We found lower odds of EoE compared with the overall findings in studies that diagnosed *H. pylori* only by gastric biopsy (OR 0.43, 95% CI 0.25 to 0.74) and in those published after 2019 (OR 0.44, 95% CI 0.28 to 0.68).

**Conclusions:**

Exposure to *H. pylori* was significantly associated with decreased odds of EoE/EE. As a stronger protective effect was found in more recent studies, the epidemiology of this association may evolve and deserve to be further monitored.

## Graphical abstract

In a large systematic review and meta‐analysis, exposure to *Helicobacter pylori* was significantly associated with decreased odds (46%) of eosinophilic oesophagitis and oesophageal eosinophilia.

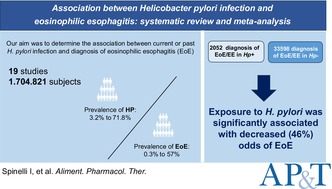

## 1 Introduction

Eosinophilic oesophagitis (EoE) is a chronic, immune‐mediated and progressive disease, often associated with atopic conditions. EoE is currently diagnosed by clinical symptoms of oesophageal dysfunction, such as dysphagia or food impaction, combined with increased eosinophilic infiltration in the oesophageal mucosa (≥ 15 eosinophils/high‐power field [HPF]), in the absence of other causes of oesophageal eosinophilia (EE) [1].

Since its earliest description by Attwood and Straumann [2, 3], EoE has been considered a rare disease. However, its prevalence and incidence are constantly increasing, with mean estimates in Western countries of 63 per 100.000 inhabitants since 2017, and incidence rates reaching up to 20 per 100,000 people per year [4, 5].

Although the aetiology of EoE is still unknown, genetic, immune and environmental factors have been associated with the pathogenesis of the disease. As with other atopic conditions, factors that trigger the T‐helper 2 (Th2)‐type immune response, such as food or aeroallergens, can be responsible for the recruitment of eosinophils in the oesophagus and the development of symptoms [6].

According to the ‘hygiene hypothesis’, the progressive industrialisation and amelioration of hygienic conditions have enhanced Th2‐predominant immune pathways and may have led, consequently, to an increase in the prevalence of allergic and atopic disorders, including EoE [7, 8, 9]. Conversely, factors eliciting Th1 response, such as infectious diseases, appear to be inversely associated with EoE [10].

*Helicobacter pylori* ( *H. pylori* ) is a Gram‐negative bacterium that is responsible for one of the most common infections worldwide [11]. It usually presents as chronic gastritis and is a known risk factor for peptic ulcer disease and gastric adenocarcinoma [11]. *H. pylori* infection is usually acquired in childhood and its prevalence is closely correlated with the socio‐economic status of the population [11].

In recent years, a progressive decline of *H. pylori* infection and a concurrent increase of EoE have been observed in Western countries, along with the improvement of socioeconomic conditions [8, 9].

As *H. pylori* triggers a Th1‐mediated immune response [12], the hypothesis of a potentially protective role of *H. pylori* towards the development of EoE has recently emerged [8, 9].

This hypothesis is supported by an increasing number of studies that observed an inverse association between *H. pylori* and EoE [9, 13], although it has not been confirmed by other studies [14]. In 2019, a systematic review and meta‐analysis of 11 studies found that exposure to *H. pylori* was associated with a 37% reduction in the odds of EoE [15]. However, the epidemiology of EoE has dramatically increased in recent years, as well as the number of pertinent studies. Therefore, our aim is to provide an updated and systematic evaluation of the association between *H. pylori* infection and EoE.

## 2 Methods

This study was conducted and reported according to the MOOSE (meta‐analyses of observational studies in epidemiology) guidelines (Table S1) [16]. Ethical approval was not required.

### 2.1 Selection Criteria

We considered eligible all studies (prospective or retrospective observational cohorts, cross‐sectional or longitudinal studies, case–control studies, clinical trials) which fulfilled the following selection criteria: (a) inclusion of paediatric or adult patients with EoE or oesophageal eosinophilia (EE); (b) investigation of *H. pylori* infection in the study population; (c) report of *H. pylori* status (positivity or negativity) in patients with and without EoE/EE. We excluded case reports, case series with fewer than 10 subjects and studies without sufficient details.

### 2.2 Information Sources, Search Strategy and Study Selection

Potentially eligible studies were identified by searching systematically MEDLINE (via PubMed), Scopus and ISI Web of Science, and without language restrictions up to 24th November 2024. The full search string is detailed in the Appendix. Moreover, the bibliographies of selected papers were manually searched to provide additional references. We did not restrict eligibility to studies published only in English. Foreign language papers were translated, where necessary. To identify potentially eligible studies published only as abstracts, conference proceedings of major conferences (Digestive Diseases Week, United European Gastroenterology Week and the Asian Pacific Digestive Week) between 2001 and 2024 were also hand‐searched. Titles and abstracts of all studies were assessed independently by two investigators (IS and SP) to exclude studies that did not meet the eligibility criteria. Any conflict was resolved by consulting a third reviewer (GI).

### 2.3 Data Extraction

Two investigators (IS and CE) extracted independently the following data from included studies, by using a standardised data extraction form: first author; year; country; study period; study design; definition of EoE/EE; protocol for endoscopic biopsies; definition of comparator; sample; population age (paediatric vs. adult population); total number of cases; *H. pylori* –positive cases; *H. pylori* –negative cases; total number of controls; *H. pylori* –positive controls; *H. pylori* –negative controls; total number of *H. pylori* –positive patients; current versus past *H. pylori* infection; and type of diagnostic testing for *H. pylori*. If the same cohort was reported in different studies, the study with the most complete dataset was chosen.

### 2.4 Quality Assessment

Two authors (CE and WF) independently assessed the quality of the included studies using the Newcastle–Ottawa Scale, a validated technique for assessing the quality of nonrandomised studies in metanalyses, with the following ranking: 1–3 points = poor quality, 4–6 points = medium quality and 7–9 points = high quality [17]. Discrepancies between reviewers regarding the collection of qualitative and quantitative data were infrequent (overall interobserver variation < 10%) and were always resolved by discussion and by consulting a third reviewer (GI).

### 2.5 Data Synthesis and Statistical Analysis

Data were analysed according to the DerSimonian and Laird method using a random‐effects model [18] and expressed in terms of odds ratio (OR) and 95% confidence intervals (CIs). Heterogeneity between study‐specific estimates was assessed using the inconsistency index (*I* ^2^) and cutoff points of < 30%, 30%–59%, 60%–75% and > 75% were considered to suggest low, moderate, substantial and considerable heterogeneities, respectively [19]. To assess the primary outcome across different subgroups of patients and to explore potential sources of heterogeneity, we performed several subgroup analyses based on the following items: study design (prospective vs. retrospective), study location (Eastern vs. Western countries), patient age (paediatrics vs. adults), diagnosis of EoE according to established criteria (presence of ≥ 15 eosinophils/HPF at oesophageal biopsies), type of diagnostic test for *H pylori*, quality of the studies (high vs. low quality), publication before versus after 2019, presence of ≥ 15 eosinophils and publication of studies after the previous metanalysis by Shah et al. [15].

Publication bias was assessed qualitatively by visual inspection of funnel plots. The analyses were performed using RevMan version 5 from the Cochrane collaboration group.

## 3 Results

### 3.1 Study Selection and Characteristics of Included Studies

**FIGURE 1:**
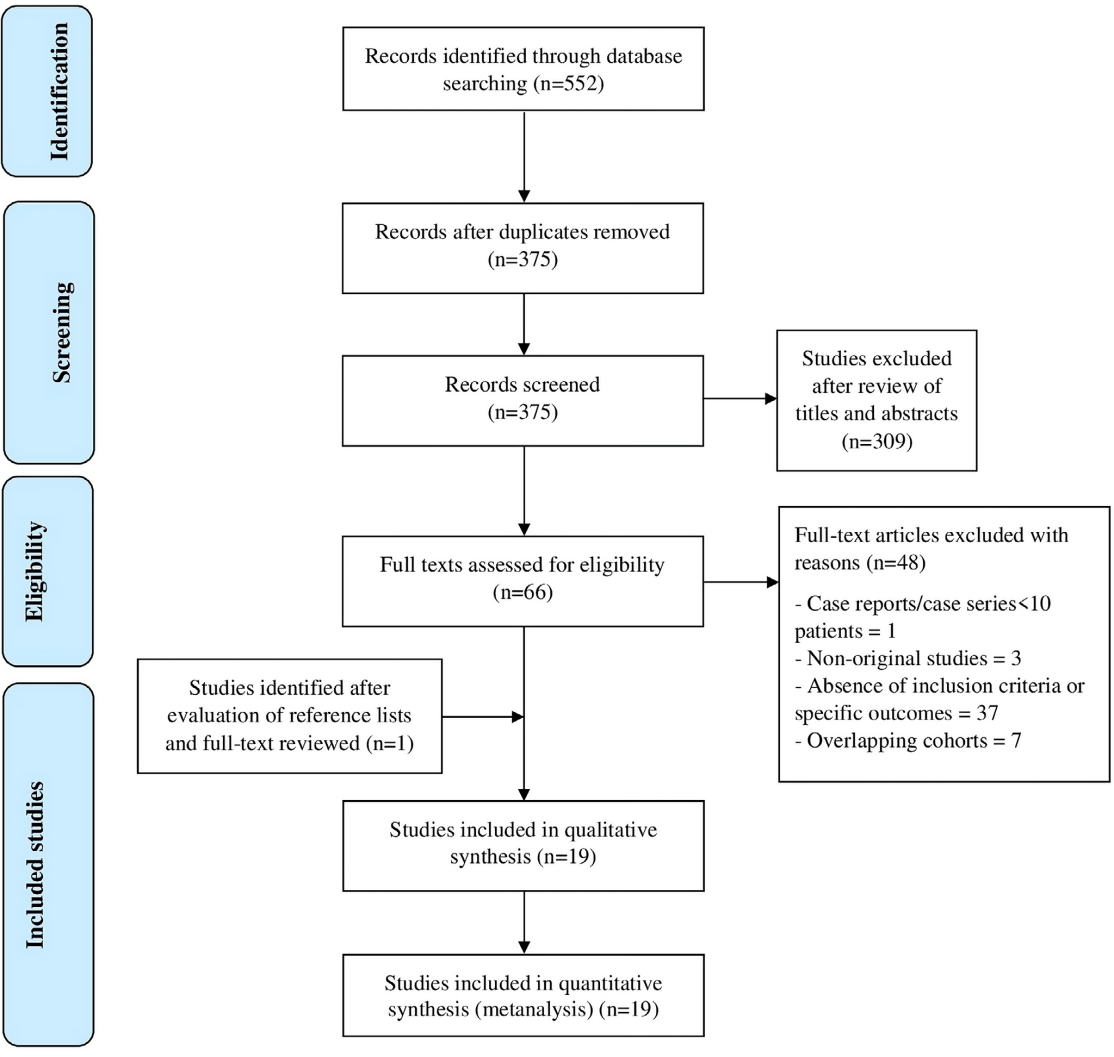
Preferred reporting items for systematic reviews and meta‐analyses (PRISMA) flow diagram of the search process.

Figure 1 shows the flow diagram of study selection. The search strategy generated 375 citations after removal of duplicates. After review of titles and abstracts, 66 of them were considered relevant and were kept for further evaluation of the full text. Eighteen studies were included in the final analysis after review of the full text [9, 13, 14, 20, 21, 22, 23, 24, 25, 26, 27, 28, 29, 30, 31, 32, 33, 34]. Moreover, after evaluating the reference lists of these articles, one additional article [35] was full‐text reviewed and added for the final analysis for a total of 19 eligible studies and 1.704.821 subjects. Characteristics of included studies are summarised in Table 1, while details of included subjects are described in Table 2. Five studies were from the United States [9, 24, 28, 32, 33], seven from Asia [21, 23, 25, 26, 29, 34, 35], five from Europe [13, 14, 27, 30, 31], one from Mexico [20], and one from Australia [22].

**TABLE 1:** Characteristics of included studies.

First author	Year	Country	Study period	Sample	Study design	Definition of EoE/EE	Biopsy protocol for EoE	Definition of comparator
Cessa‐Zanatta et al. [20]	2024	Mexico	2016–2021	190	Prospective, case–control, single centre	Oesophageal dysfunction and > 15 eos/HPF	6 oesophageal biopsies	< 15 eos/HPF
Chang et al. [21]	2023	Korea	2003–2022	135	Retrospective, case–control, single centre	Oesophageal dysfunction and > 15 eos/HPF	Oesophageal biopsies	No significant gastrointestinal disease at endoscopic evaluation
Cheung et al. [22]	2003	Australia	1989–2000	42	Retrospective, cohort, single centre	Dysphagia and > 20 eos/HPF	Oesophageal biopsies	Dysphagia and ≤ 5 eos/HPF
Dellon et al. [9]	2011	United States	2008–2010	165,017	Retrospective, case–control, multicentre (pathology database)	EE: ≥ 15 eos/HPF; EoE: EE + clinical suspicion for EoE and no reflux or BE	Oesophageal biopsies	< 15 eos/HPF
Dolstra et al. [23]	2023	Israel	2017–2021	558	Retrospective, case–control, single centre	Accepted diagnostic criteria of EoE	Oesophageal biopsies	Celiac disease or IBD
Elitsur et al. [24]	2014	United States	2007–2012	966	Retrospective, case–control, single centre	≥ 15 eos/HPF	6 oesophageal biopsies	< 15 Eos/HPF
Furuta et al. [25]	2013	Japan	2010–2011	72	Retrospective, case–control, single centre	Oesophageal dysfunction and ≥ 15 eos/HPF	Not stated	Matched controls without EoE
Imamura et al. [26]	2020	Japan	2012–2018	252	Retrospective, case–control, multicentre	≥ 15 eos/HPF	Not stated	EoE absent
Lee et al. [35]	2020	Korea	2003–2020	117	Retrospective, case control, single centre	≥ 15 eos/HPF	Not stated	Matched controls without EoE
Lluncor‐Salazar et al. [27]	2018	Spain	2000–2014	61	Retrospective, cross‐sectional, single centre	≥ 15 eos/HPF	Not stated	Proton pump inhibitor–responsive oesophageal eosinophilia
Low et al. [28]	2023	United States	1999–2018	936,399	Retrospective, cohort	Not stated	Not stated	Not stated
Ma et al. [29]	2015	China	Not stated	1021	Prospective, cross‐sectional	EE: > 15 eos/HPF	≥ 4 oesophageal biopsies	0 eos/HPF
Molina‐Infante et al. [13]	2018	Spain, Italy, France, Colombia	2014–2017	808	Prospective, case–control, multicentre	Oesophageal symptoms and ≥ 15 eos/HPF	≥ 6 oesophageal biopsies	Oesophageal symptoms and < 5 eos/HPF
Norder Grusell et al. [30]	2018	Sweden	2009–2014	27	Prospective, cohort, single centre	Oesophageal dysfunction and ≥ 15 eos/HPF	Oesophageal biopsies	GERD (typical symptoms + endoscopic and/or histopathologic esophagitis)
Ronkainen et al. [31]	2007	Sweden	1998	1000	Prospective, cross‐sectional, single centre	EE: > 0 eos/HPF	≥ 2 oesophageal biopsies	0 eos/HPF
Sealock et al. [32]	2013	United States	Not stated	1357	Prospective, cross‐sectional, single centre	EE: > 15 eos/HPF; EoE (definite): EE + oesophageal symptoms + acid suppression meds EoE (probable): EE + either oesophageal symptoms or acid suppression meds	≥ 1 oesophageal biopsy	≤ 15 eos/HPF
Sonnenberg et al. [33]	2017	United States	2008–2015	596,479	Retrospective, case–control, multicentre (pathology database)	3 definitions, variable certainty: (a) > 15 eos/HPF + dysphagia (b) > 15 eos/HPF + dysphagia and exclusion of GERD/reflux esophagitis, BE, eosinophilic gastroenteritis, IBD or other aetiologies for eosinophilia (c) > 50 eos/HPF and exclusion of other causes listed in (b)	Oesophageal biopsies	No histological abnormalities
Suzuki et al. [34]	2022	Japan	2010–2019	146	Retrospective, case control, single centre	Oesophageal symptoms and ≥ 15 eos/HPF	≥ 2 oesophageal biopsies	≥ 15 eos/HPF without symptoms
von Arnim et al. [14]	2016	Germany	Not stated	174	Retrospective, case–control, single centre	Oesophageal symptoms and > 15 eos/HPF	4 oesophageal biopsies	Matched controls without EoE

Abbreviations: BE, Barrett oesophagus; EE, oesophageal eosinophilia; EoE, eosinophilic esophagitis; eos, eosinophils; GERD, gastroesophageal reflux disease; HPF, high‐power field; IBD, inflammatory bowel disease.

**TABLE 2:** Characteristics of included subjects.

First author	Paediatric vs. adult patients	Mean age (years)	Males	Total EoE cases	Hp pos cases	Hp neg cases	Total controls	Hp pos controls	Hp neg controls	Total Hp patients	Current vs. former infection	Hp testing
Cessa‐Zanatta et al. [20]	Adult	Cases: 40.39 ± 15.56 Controls: 43.62 ± 14.22	Cases: 65.8% Controls: 51.3%	38	14/38 (36.8%)	24/38 (63.2%)	152	107/152 (70.4%)	45/152 (29.6%)	121/190 (63.6%)	Current	Gastric biopsy
Chang et al. [21]	Adult	Cases: 19.3 ± 23.9 Controls: 20.3 ± 21.9	Cases: 66.7% Controls: 66.7%	45	2/45 (4.4%)	43/45 (95.6%)	90	17/90 (18.9%)	73/90 (81.1%)	19/135 (14%)	Current	Rapid urease test, UBT, gastric biopsy
Cheung et al. [22]	Paediatric	Cases: 10.1 ± 4.0 Controls: 8.3 ± 4.7	Cases: 76% Controls: 52%	21	1/21 (4.8%)	20/21 (9.5%)	21	2/21 (9.5%)	19/21 (90.5%)	3/42 (7.1%)	Not stated	Not stated
Dellon et al. [9]	Both (paediatric: 2.1%)	55.8 ± 16.2	46.1%	EE: 5767 EoE: 2367	EE: 326/5767 (5.7%) EoE: 121/2367 (5.1%)	EE: 5441/5767 (94.3%) EoE: 2246/2367 (94.9%)	EE: 56301 EoE: 56301	4048/56301 (7.2%)	52,253/56301 (92.8%)	EE: 4374/62068 (7.0%) EoE: 4169/58668 (7.1%)	Not stated	Gastric biopsy
Dolstra et al. [23]	Paediatric	Cases: 12.3 Controls: not stated	Cases: 66.7% Controls: not stated	41	6/41 (14.6%)	35/41 (85.4%)	517	114/517 (22%)	403/517 (78%)	120/558 (18%)	Current	Gastric biopsy and/or a positive Hp culture
Elitsur et al. [24]	Paediatric	12.0 ± 3.3	50%	62	1/62 (1.6%)	61/62 (98.4%)	904	30/904 (3.3%)	874/904 (96.7%)	31/966 (3.2%)	Not stated	Rapid urease test, gastric biopsy
Furuta et al. [25]	Not stated	Cases: 50.9 ± 17.4 Controls: 50.5 ± 16.5	Cases: 61.1% Controls: 61.1%	18	4/18 (22.2%)	14/18 (77.7%)	54	30/54 (55.6%)	24/54 (44.4%)	34/72 (47.2%)	Not stated	Hp serology
Imamura et al. [26]	Both	Cases: 45.2 Controls 47.1	Cases: 68.2% Controls: 67.7%	66	7/66 (10.6%)	59/66 (89.4%)	186	34/186 (18.3%)	152/186 (81.7%)	41/252 (16.2%)	Not stated	Hp serology, UBT, gastric biopsy
Lee et al. [35]	Both	Cases: 18.1 Controls: not stated	Cases: 64.1% Controls: not stated	39	2/39 (5.1%)	37/39 (95%)	78	17/78 (21.8%)	61/78 (78.2%)	19/117 (16.2%)	Current	Rapid urease test or gastric biopsy
Lluncor‐Salazar et al. [27]	Adult	34.6 ± 16.4	75.4%	35	10/35 (28.6%)	25/35 (71.4%)	26	8/26 (30.8%)	18/26 (69.2%)	18/61 (29.5%)	Not stated	Not stated
Low et al. [28]	Adult	58.4 ± 15.0	90.7%	2861	288/2861 (10%)	2573/2861 (90%)	933,538	218,475/933538 (23.4%)	715,063/933538 (76.6%)	218,763/936399 (23.3%)	Current	Hp serology or gastric biopsy
Ma et al. [29]	Adult	50.6 ± 12.2	55.2%	67	46/67 (68.7%)	21/67 (31.3%)	954	687/954 (72.0%)	267/954 (28.0%)	733/1021 (71.8%)	Not stated	Hp serology
Molina‐Infante et al. [13]	Both	Cases: 35 Controls: 37	Cases: 74% Controls: 71%	404	151/404 (37.4%)	253/404 (62.6%)	404	161/404 (39.9%)	243/404 (60.1%)	312/808 (38%)	Not stated	Rapid urease test, UBT, gastric biopsy, antigen stool test
Norder Grusell et al. [30]	Adult	44 ± 12.2	80%	9	0/9 (0.0%)	9/9 (88.9%)	14	2/14 (14.3%)	12/14 (85.7%)	2/23 (8.7%)	Not stated	Rapid urease test
Ronkainen et al. [31]	Adult	53.5	48.8%	EE, 48	8/48 (1.7%)	40/48 (83.3%)	952	331/952 (34.8%)	621/952 (65.2%)	339/1000 (33.9%)	Not stated	Gastric biopsy and Hp culture
Sealock et al. [32]	Adult	61.5	Not stated	33	3/31 (9.7%)	28/31 (90.3%)	1324	285/1250 (22.8%)	965/1250 (77.2%)	288/1281 (22.5%)	Not stated	Hp serology or gastric biopsy
Sonnenberg et al. [33]	Both	Cases: 46.0 Controls: 55.7	Cases: 62.8% Controls: 37.0%	25,969	1156/25969 (4.5%)	24,813/25969 (95.5%)	284,552	20,683/284552 (7.3%)	263,869/284552 (92.7%)	21,839/310521 (7.0%)	Not stated	Gastric biopsy
Suzuki et al. [34]	Adult	49.4 ± 11.5	77.4%	71 (EoE)	19/71 (26.5%)	52/71 (73.5%)	75 (EE)	16/75 (21.3%)	59/75 (78.7%)	35/146 (24%)	Both	Hp serology, UBT, antigen stool test
von Arnim et al. [14]	Adult	36.5	81%	58	8/58 (13.8%)	50/58 (86.2%)	116	44/116 (37.9%)	72/116 (62.0%)	52/174 (29.9%)	Both	Hp serology

Abbreviations: EE, oesophageal eosinophilia; EoE, eosinophilic esophagitis; Hp, *Helicobacter pylori* ; neg, negative; pos, positive; UBT, urea breath test.

Thirteen studies were retrospective [9, 14, 21, 22, 23, 24, 25, 26, 27, 28, 33, 34, 35], and six studies were prospective [13, 20, 29, 30, 31, 32]. When stated, 10 studies had a case–control design [9, 13, 14, 20, 21, 24, 25, 26, 33, 35] and 7 studies were cross‐sectional [27, 29, 31, 32] or cohort studies [22, 28, 30]. When detailed, studies were conducted between 1989 and 2021. Study samples ranged between 27 [30] and 936,399 subjects [28]. Both adults and paediatrics were recruited in five studies [9, 13, 26, 33, 35], while only paediatric patients were included in three studies [22, 23, 24], and only adult patients in 11 studies [14, 20, 21, 25, 27, 28, 29, 30, 31, 32, 34], respectively. Agreement between investigators for assessment of study eligibility was excellent (κ statistic = 0.87). Two studies [22, 30] were of medium quality, while all others were judged as high quality (Tables S2–S4).

### 3.2 EoE‐Related Characteristics

Seventeen studies (89%) included patients with EoE. EoE was diagnosed by the presence of ≥ 15 eosinophils/HPF at oesophageal biopsies plus relevant symptoms in 11 studies [9, 13, 14, 20, 21, 22, 25, 29, 30, 32, 33, 34], by the presence of ≥ 15 eosinophils/HPF at oesophageal biopsies regardless of symptoms in five studies [24, 26, 27, 35, 36] and according to the accepted diagnostic criteria for EoE in another study [23]. Moreover, the EoE definition was not reported in another study [28].

Five studies included patients with EE [9, 29, 31, 32, 33]. EE was diagnosed by the presence of ≥ 15 eosinophils/HPF at oesophageal biopsies without symptoms in three studies that included also patients with EoE, defined as the presence of ≥ 15 eosinophils/HPF at oesophageal biopsies plus relevant symptoms [9, 29, 32]. The two remaining studies included only patients with EE, which was diagnosed by the presence of ≥ 15 eosinophils/HPF at oesophageal biopsies plus relevant symptoms in one of them [33] and by the presence of ≥ 0 eosinophils/HPF at oesophageal biopsies in another one [31].

At least six oesophageal biopsies were used to diagnose EoE in three studies [13, 20, 24], while at least one biopsy, two biopsies and four biopsies were collected in one study [32], two studies [31, 34] and two studies [14, 29], respectively. The number of biopsies was not described in six studies [9, 21, 22, 23, 30, 33], and the biopsy protocol was not detailed in five studies [25, 26, 27, 28, 35]. Patients with a diagnosis of EoE ranged from 0.3% [28] to 57% [27] in different cohorts.

The comparator population was defined according to histopathological features in 11 studies (58%). Specifically, a cut‐off of < 15 eosinophils/HPF was chosen in four studies [9, 20, 24, 32], while a cut‐off of less than five eosinophils/HPF in the presence of oesophageal symptoms was put in two studies [13, 22], and four studies used the absence of eosinophils or histological abnormalities as comparator [26, 29, 31, 33].

Finally, one study selected patients with EE responsive to proton pump inhibitors as the comparator group [27]. In three studies, symptom‐ or endoscopy‐related features were used to select controls, including the absence of symptoms in patients with ≥ 15 eosinophils/HPF [34], the presence of gastroesophageal reflux disease, defined as typical symptoms with endoscopic and/or histopathologic oesophagitis [30], and the absence of gastrointestinal disorders at endoscopic evaluation [21].

Finally, the comparator group included matched controls without a history of EoE in three studies [14, 25, 35], and patients with celiac disease or inflammatory bowel disease (IBD) in one study [23], while it was not defined in one study [28].

### 3.3 *H. pylori* –Related Characteristics

The overall prevalence of *H. pylori* infection across studies was 18%, ranging between 3.2% [24] and 71.8% [29], *H. pylori* prevalence was 43% in Eastern countries [21, 23, 25, 26, 29, 34, 35] and 14% in Western countries [9, 13, 14, 20, 22, 24, 27, 28, 30, 31, 32, 33], respectively.

*H. pylori* was diagnosed by gastric biopsy and histology in most studies (12 studies, 63%) [9, 13, 20, 21, 23, 24, 26, 28, 31, 32, 33, 35], alone [9, 20, 33] or using other testing methods as alternatives, including *H. pylori* culture [23, 31], rapid urease test [13, 21, 24, 35], urea breath test [13, 21, 26], *H. pylori* serology [26, 28, 32] or *H. pylori* antigen stool test [13]. Rapid urease test was the only method of diagnosis in one study [30], while three studies used only *H. pylori* serology [14, 25, 29] and noninvasive methods (urea breath test, *H. pylori* serology or antigen stool test) were only used in another study [34]. Finally, the diagnostic testing for *H. pylori* was not stated in two studies [22, 27]. Five studies enrolled patients with current *H. pylori* infection [20, 21, 23, 28, 35], one study enrolled patients with either current infection or past infection [34], while in 13 studies [9, 13, 14, 22, 24, 25, 26, 27, 29, 30, 31, 32, 33], the discernment between current and past infection was not available.

### 3.4 *H. pylori* Exposure and Odds of EoE

**FIGURE 2:**
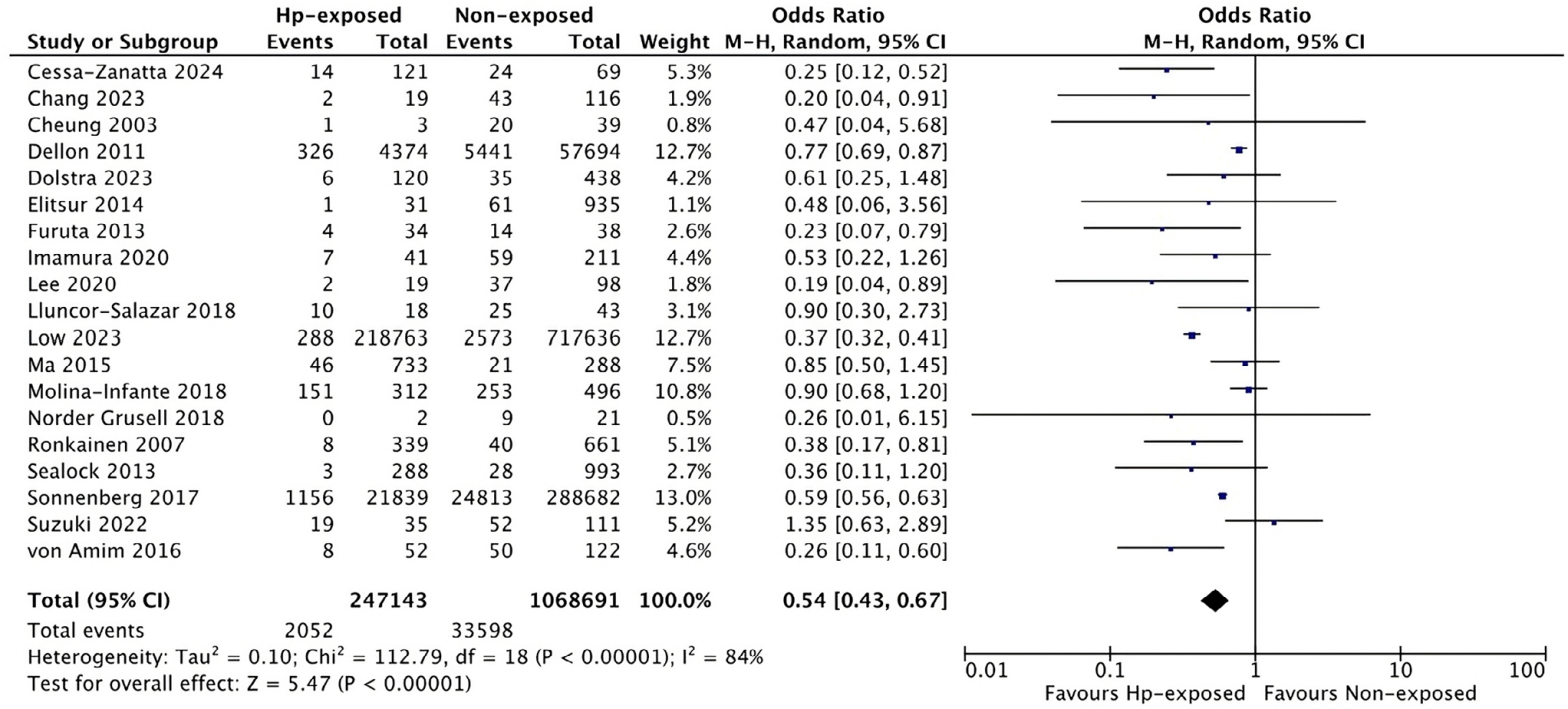
Overall risk of EoE in patients exposed to *Helicobacter pylori* and without a history of *H. pylori* exposure.

As the definitions of EoE and EE often overlapped among included studies, we combined findings between these two diseases and performed pertinent subgroup analyses. Overall, based on 19 studies, EoE/EE was diagnosed in 2052 patients exposed to *H. pylori* and 33,598 patients without a history of *H. pylori* exposure. More specifically, *H. pylori* infection was associated with 46% lower odds of EoE/EE (OR: 0.54, 95% CI 0.43 to 0.67), with high heterogeneity (*I* ^2^ = 84%, *p* < 0.00001) (Figure 2). When we limited the analysis to the 16 studies that had defined EoE as the presence of ≥ 15 eosinophils/HPF at oesophageal biopsies [9, 13, 14, 20, 21, 22, 24, 25, 26, 27, 29, 30, 32, 33, 34, 35], this result was confirmed (OR 0.54, 95% CI 0.40 to 0.74), although with lower heterogeneity (*I* ^2^ = 59%, *p* = 0.003) (Figure S1). Moreover, we analysed only studies including patients with EE, with slight differences compared with overall findings (OR 0.65, 95% CI 0.52 to 0.82, Figure S2).

As shown in Figure S3, the odds of EoE in *H. pylori* –exposed subjects were also similar when subgrouping Western cohorts (*n* = 12 studies; OR 0.52, 95% CI 0.41 to 0.67, *I* ^2^ = 89%, *p* < 0.00001) [9, 13, 14, 20, 22, 24, 27, 28, 30, 31, 32, 33] and Eastern studies (*n* = 7 studies; OR 0.53, 95% CI 0.30 to 0.90, *I* ^2^ = 52%, *p* = 0.05) [21, 23, 25, 26, 29, 34, 35], as well as by analysing separately prospective studies (*n* = 6 studies; OR 0.52, 95% CI 0.31 to 0.88, *I* ^2^ = 67%, *p* = 0.009) [13, 20, 29, 30, 31, 32] and retrospective cohorts (*n* = 13 studies; OR 0.53, 95% CI 0.40 to 0.69, *I* ^2^ = 87%, *p* < 0.00001), as summarised in Figure S4 [9, 14, 21, 22, 23, 24, 25, 26, 27, 28, 33, 34, 35]. Also, a subgroup analysis excluding two medium‐quality studies [22, 30] did not exert different results (OR 0.54, 95% CI 0.43 to 0.68, *I* ^2^ = 86%, *p* < 0.00001, Figure S5).

**FIGURE 3:**
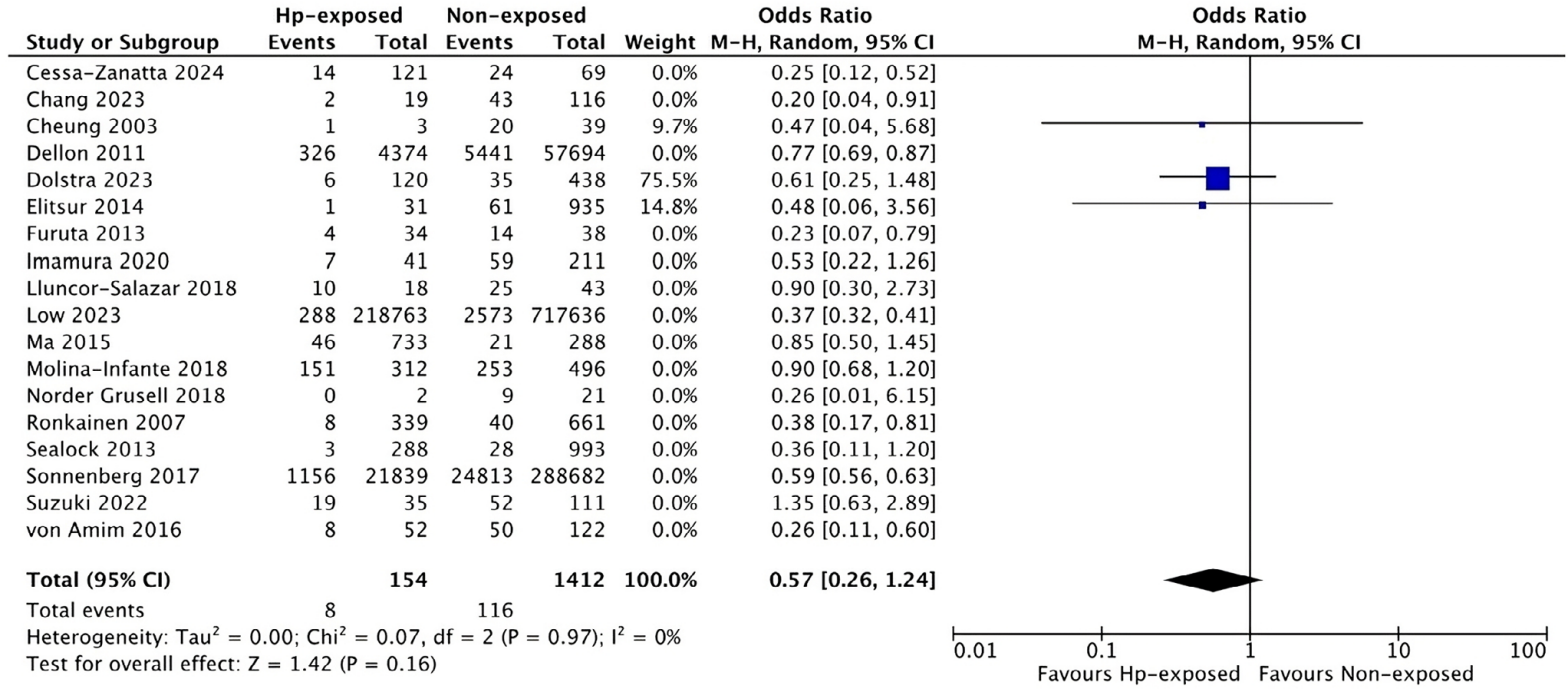
Risk of EoE in adult and paediatric patients, respectively, exposed to *Helicobacter pylori* and without a history of *H. pylori* exposure.

Interestingly, we did not observe a significant odds reduction for EoE in paediatric patients exposed to *H. pylori* based on three studies (OR 0.57, 95% CI 0.26 to 1.24, *I* ^2^ = 0%, *p* = 0.97) [22, 23, 24], while slightly lower *H. pylori* –associated odds for EoE were found in adults (*n* = 11 studies, OR 0.46, 95% CI 0.31 to 0.67, *I* ^2^ = 64%, *p* < 0.003) [14, 20, 21, 25, 27, 28, 29, 30, 31, 32, 34], as represented in Figure 3.

In six studies that diagnosed *H. pylori* only by gastric biopsy [9, 20, 23, 31, 32, 35], lower odds of EoE, compared with the overall findings were observed (OR 0.43, 95% CI 0.25 to 0.74, *I* ^2^ = 70%, *p* = 0.006, Figure S6), while a nonsignificant protection against EoE was found in three studies using only serology [14, 25, 29] to diagnose *H. pylori* (OR: 0.41, 95% CI 0.16 to 1.04, *I* ^2^ = 74%, *p* = 0.02, Figure S7).

**FIGURE 4:**
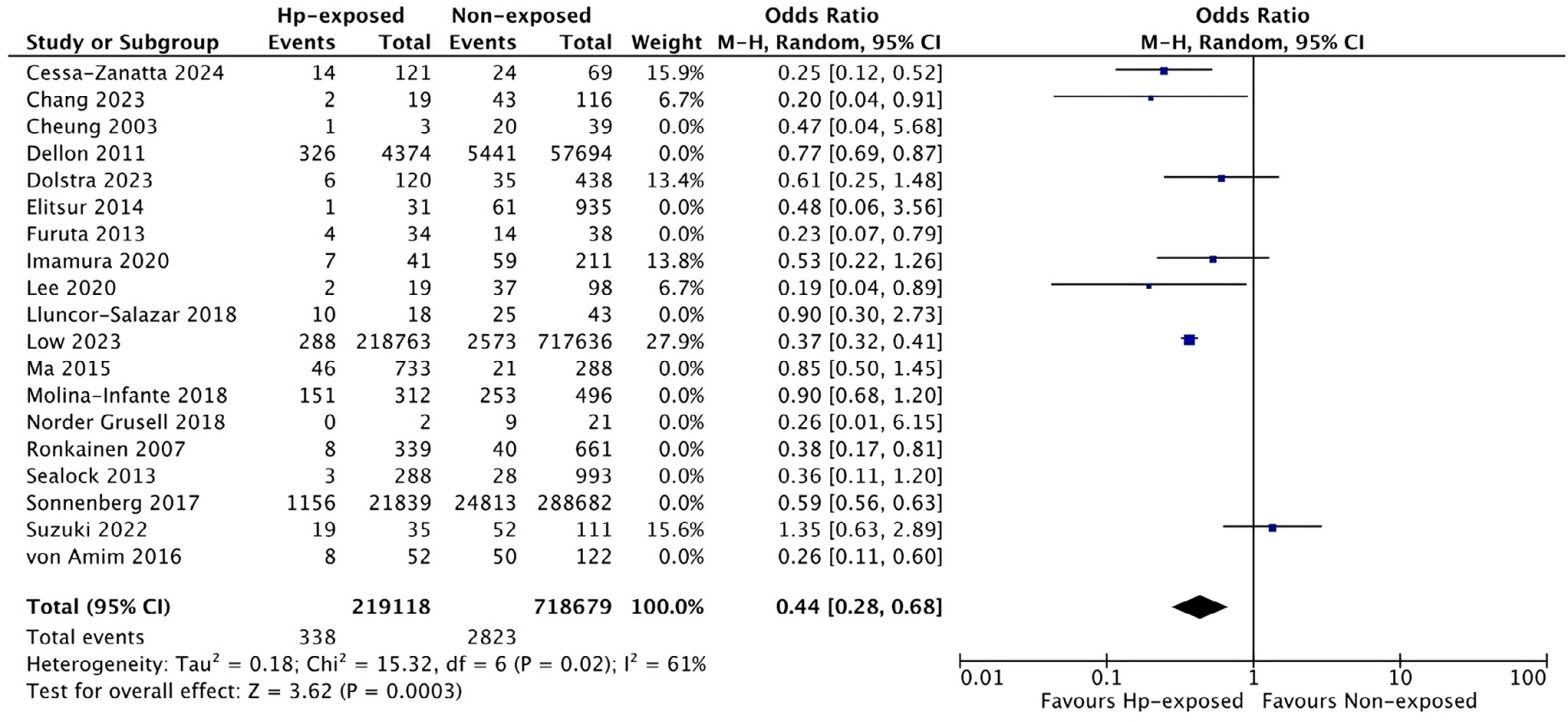
Risk of EoE in subjects exposed to *Helicobacter pylori* and without a history of *H. pylori* exposure based on studies published before or after 2019, respectively.

Finally, we also evaluated studies published after the previous meta‐analysis by Shah et al. [15] Notably, we found even further decreased odds for EoE in these more recent studies, published after 2019 (*n* = 7 studies, OR 0.44, 95% CI 0.28 to 0.68, *I* ^2^ = 61%, *p* = 0.02) compared with those published by 2019 (*n* = 12 studies, OR 0.64, 95% CI 0.53 to 0.79, *I* ^2^ = 67%, *p* = 0.0005), as summarised in Figure 4.

## 4 Discussion

The number of EoE diagnoses has considerably risen in the last decades, with a more pronounced rise in adults than in children. This increase can be attributed both to higher prevalence and incidence and to a heightened awareness of this condition among patients and physicians [36, 37].

Due to such epidemiological growth, EoE now represents a considerable financial burden, as patients with EoE incur significantly higher monthly healthcare resource utilisation in terms of inpatient visits, emergency department visits, and outpatient visits versus matched controls [38]. In the United States, EoE was associated with estimated annual costs of $1.3 billion in 2024 [39]. Therefore, the understanding of EoE pathogenesis and related risk/protective factors is crucial to improving its management and establishing preventive strategies. Among the protective factors, *H. pylori* infection has been advocated in several studies, but conflicting data are available and the epidemiology of EoE is rapidly changing. Thus, we decided to conduct a systematic review and meta‐analysis to provide an updated association between EoE and *H. pylori* infection, pooling together data from 19 studies and 1.704.821 subjects.

In our study, current or past *H. pylori* infection was associated with 46% lower odds of EoE/EE.

Our results differ slightly from those found in a previous meta‐analysis [15], where the *H. pylori* –associated odds reduction was 37% for EoE and 38% for EE, respectively. Several reasons may explain this discrepancy. First, Shah et al. analysed 11 studies [15], while we included a total of 19 cohorts in our study. As the number of studies in our meta‐analysis almost doubled compared with those included in the previous one, with a considerable increase in the number of patients (937.858 newly added patients), our results are also expected to differ from the previous ones. However, another explanation might lie in the rapidly evolving epidemiology of EoE: The increased diffusion of this condition may allow us to identify risk factors and protective factors in a more robust way than in the past, especially with a highly common infection such as *H. pylori* . To corroborate this finding, we also performed a subgroup analysis of studies published after the previous metanalysis [15], and found that the inverse association between *H. pylori* and EoE was even stronger than in the overall population, with a 56% odds reduction, that is, a 19% absolute odds decrease compared with the previous study [15]. This specific finding might suggest that the association between EoE and *H. pylori* is still far from reaching a steady state and deserves to be monitored with further studies in the future. Specific factors, like the rapidity of EoE in reaching its epidemiological plateau and the potential decrease of *H. pylori* diffusion in the future (as based on the test‐and‐treat strategy promoted by the Maastricht VI guidelines that recommend eradicating the infection whenever detected [11]) are expected to influence the robustness of this association.

As per our current data, the protective effect of *H. pylori* against the risk of EoE appears not to be influenced by geographical location, as subgroup analysis for Eastern versus Western countries did not show any substantial difference from overall results. Our findings were comparable between Eastern and Western studies despite the large difference in their prevalence of *H. pylori* infection (in line with available literature) [40], supporting the strength of this association and the generalisability of our findings. A likely rationale behind this observation is the economic status of countries that hosted included studies. Regardless of their geographical location, included studies come mainly from high‐income countries [41], suggesting a comparable level of improved socioeconomic status, industrialisation and sanitation, which influence the risk of EoE according to the ‘hygiene hypothesis’ [7, 8, 9].

Interestingly, we did not observe a significant odds reduction for EoE in paediatric patients exposed to *H. pylori* . One plausible reason relies on the limited number of studies conducted in paediatric cohorts (*n* = 3), but we may also hypothesise that very early‐onset EoE (V‐EoE) is more strongly influenced by genetic and early‐life factors than EoE with adult onset, similar to other complex immune‐mediated diseases, such as monogenic very early‐onset IBD (VEO‐IBD) [42]. This hypothesis is also supported by a recent study of 57 patients with V‐EoE that identified caesarean delivery and CAPN14 genetic variation as promoters of earlier disease development [43]. However, this finding may also depend on the small sample size/number of available studies. Unfortunately, the included studies did not report the outcomes stratified according to the median age, so a specific subgroup analysis based on this parameter was unfeasible. This is a limitation of our study, and further evidence is needed to clarify the impact of *H. pylori* infection on EoE in paediatrics.

The study design of included studies appeared not to influence our results, as findings observed in prospective studies and retrospective cohorts were also highly similar. This result may also have a pertinent explanation, as most included studies (90%) were of high quality, and this factor might be a surrogate of the reliability of retrieved data, also in retrospective studies.

Conversely, our findings differed significantly according to the type of diagnostic testing used to detect *H. pylori* , as we observed lower odds of EoE (57% reduction) in studies where *H. pylori* was diagnosed only by gastric biopsy, while the protective effect of *H. pylori* was not significant in studies using only serology to diagnose *H. pylori* . Although we were not able to separate results between current and past infection in our study, these findings might support a major role of active *H. pylori* presence rather than previous exposure, as serology cannot discriminate the status of infection (current vs. past infection) [11]. However, our findings should be taken with caution, as only three studies used serology as a unique diagnostic test.

Overall, our results are supported by a strong biological background. *H. pylori* has been inversely associated with allergic comorbidities such as asthma, allergic rhinitis and atopic dermatitis [44, 45] and was shown to be a protective factor against asthma in experimental models [46]. Since EoE shares the same Th2‐mediated pathogenesis, our findings appear consistent with this line of evidence. While allergic diseases are driven by a Th2 immune response, infectious processes, such as *H. pylori*, are characterised by a Th1 cell response. Originating from a Th2‐dominated prenatal environment, in the absence of external stimulation by microbial components that elicit Th1 responses, the immune system of a newborn fails to shift from a Th2 to a Th1 balance, leading to a Th2‐dominated immune profile and an increased risk of developing allergic diseases and, consequently, EoE [47].

Another explanation of the potential protective role of *H. pylori* against EoE could lie in the decreased oesophageal acid load associated with chronic *H. pylori* infection, as already observed with gastroesophageal reflux disease: [48] As the acid reflux may damage the oesophageal barrier and let allergens elicit a Th2 response, thus triggering EoE, its decrease may be protective against the development of this condition.

However, *H. pylori* infection may also be a general marker of poor hygiene conditions [49], making the inverse association with EoE a proxy for the hygiene hypothesis.

Our metanalysis presents some limitations. The high heterogeneity among the included studies, particularly pronounced when examining the overall incidence of EoE/EE in patients exposed to *H. pylori* compared with those unexposed, suggests caution in the interpretation of our findings.

Notably, we did not perform any separate analysis for EE, as previously done [15]. However, we chose this strategy because in all three studies that included patients with EE [9, 29, 32] it was defined by the presence of ≥ 15 eosinophils/HPF at oesophageal biopsies. As this cut‐off already defines the accepted diagnosis for EoE, we assume that the two conditions overlap, without any potential bias in interpreting results. Another potential limit of our study was the absence of data from low‐income countries and specific regions, for example, Africa, South America or Southeast Asia. Pertinent studies are advocated not only to evaluate the association between *H. pylori* and EoE but, more widely, to give insights into the epidemiology of this condition in these countries. Also, our data did not allow us to discriminate between current and past *H. pylori* infection in most studies; therefore, we were not able to evaluate the influence of eradication therapies on the association between *H. pylori* and EoE. Finally, the wide differences in *H. pylori* testing strategies among included studies may contribute to false‐negative diagnoses, potentially biasing our results.

In conclusion, in this updated, large systematic review and meta‐analysis of 19 studies and > 1.700.000 subjects, we found an inverse association between exposure to *H. pylori* and EoE/EE, with a 46% odds reduction. As we found a stronger signal (56% reduction) in studies published after 2019 and not included in the previous meta‐analysis, the epidemiology of this association may evolve and deserves to be monitored in the future, as well as to be corroborated by further and well‐designed population studies.

## Author Contributions

**Irene Spinelli:** data curation, writing – original draft, writing – review and editing, investigation. **Serena Porcari:** methodology, investigation, writing – original draft, writing – review and editing, data curation, supervision. **Chiara Esposito:** investigation, writing – original draft, data curation. **William Fusco:** data curation. **Francesca Romana Ponziani:** visualization, writing – review and editing. **Cristiano Caruso:** visualization, writing – review and editing. **Edoardo Vincenzo Savarino:** writing – review and editing, visualization. **Antonio Gasbarrini:** visualization, writing – review and editing. **Giovanni Cammarota:** visualization, writing – review and editing. **Marcello Maida:** methodology, writing – review and editing, visualization, formal analysis. **Antonio Facciorusso:** formal analysis, writing – review and editing, visualization, methodology. **Gianluca Ianiro:** conceptualization, investigation, writing – original draft, writing – review and editing, methodology, visualization, supervision, data curation.

## Conflicts of Interest

F.R.P. has received speaker fees, advisory board fees and travel grants from Bayer, MSD, Roche, Eisai, Ipsen, AstraZeneca, Gilead, Abbvie and Alfasigma. C.C. has received research support from GSK and AstraZeneca. E.V.S. has served as a speaker for Abbvie, Agave, AGPharma, Alfasigma, Aurora Pharma, CaDiGroup, Celltrion, Dr. Falk, EG Stada Group, Fenix Pharma, Fresenius Kabi, Galapagos, Janssen, JB Pharmaceuticals, Innovamedica/Adacyte, Malesci, Mayoly Biohealth, Omega Pharma, Pfizer, Reckitt Benckiser, Sandoz, SILA, Sofar, Takeda, Tillots and Unifarco; has served as a consultant for Abbvie, Agave, Alfasigma, Biogen, Bristol‐Myers Squibb, Celltrion, Diadema Farmaceutici, Dr. Falk, Fenix Pharma, Fresenius Kabi, Janssen, JB Pharmaceuticals, Merck & Co, Reckitt Benckiser, Regeneron, Sanofi, SILA, Sofar, Synformulas GmbH, Takeda and Unifarco; and he received research support from Pfizer, Reckitt Benckiser, SILA, Sofar, Unifarco and Zeta Farmaceutici. AG has served as a consultant for Eisai S.r.l., 3PSolutions, Real Time Meeting, Fondazione Istituto Danone, Sinergie S.r.l., Board MRGE and Sanofi S.p.A.; as a speaker for Takeda S.p.A., AbbVie and Sandoz S.p.A.; and as an advisory board member for VSL3 and Eisai. M.M. has served as a consultant for Norgine and received speaker fees and/or travel grants from Norgine, Fujifilm, Aurora Biofarma and Malesci. G.I. has served as a speaker for Alfa Sigma, Biocodex, Illumina, Malesci, Sofar and Tillotts Pharma, and as a consultant/advisor for Biocodex, Malesci and Tillotts Pharma. The other authors have no potential competing interests to disclose.

## Authorship

*Guarantor of article*: Gianluca Ianiro.

## Supporting information


Data S1.

## Data Availability

The data that support the findings of this study are available from the corresponding author upon reasonable request.
